# Examining the Effects of Dual and Single Task Exercises in Individuals with Type 2 Diabetes: A Randomized Controlled Trial

**DOI:** 10.3390/jcm15072761

**Published:** 2026-04-06

**Authors:** Sidrenur Aslan Kolukisa, Ferruh Taspinar, Betul Taspinar

**Affiliations:** 1 Department of Occupational Therapy, Vocational School of Health Services, Artvin Coruh University, Artvin 08000, Turkey; 2 Department of Physiotherapy and Rehabilitation, Faculty of Health Sciences, Izmir Democracy University, Izmir 35290, Turkey

**Keywords:** cognitive functions, exercise, postural balance, type 2 diabetes mellitus

## Abstract

**Background**: Complications developing in individuals with Type 2 Diabetes Mellitus (T2DM) lead to functional impairments and losses in postural balance; however, changes in cognitive functions are also observed and are often overlooked. Dual-task exercises allow simultaneous engagement of balance and cognitive functions. Therefore, this study aimed to investigate the effects of dual-task exercise training on cognitive functions, balance, and functional status in individuals with T2DM. **Methods**: In this study, 40 individuals diagnosed with T2DM were randomly assigned to three groups: the dual-task exercise group (DTEG, *n* = 13), the single-task exercise group (STEG, *n* = 13), and the control group (CG, *n* = 14). Over eight weeks, balance exercises were administered to the STEG, while simultaneous balance and cognitive exercises were applied to the DTEG, twice weekly under the supervision of a physiotherapist. Participants in the control group received no intervention. Dual-task performance, cognitive functions, balance, and functional status were assessed at baseline and at the end of eight weeks. Dual-task performance was defined as the primary outcome. **Results**: After the intervention, for the primary outcome, dual-task performance (TUG single-task condition and TUG dual-task condition), both exercise groups showed greater improvements than controls. Both exercise groups also demonstrated significant improvements in balance, functional status, and cognitive outcomes compared to the control group. In the between-group comparisons, both exercise groups showed significant improvements in several cognitive functions compared with the control group (*p* < 0.05). In addition, the MoCA total score was significantly higher in the DTEG compared with the other groups. **Conclusions**: Both dual-task and single-task exercises improve cognitive function, balance, and functional status in individuals with T2DM.

## 1. Introduction

Type 2 diabetes mellitus (T2DM) is a multifactorial metabolic disorder resulting from the interaction between genetic predisposition and environmental and lifestyle factors, affecting individuals across all age groups and both sexes [1]. It is the most common form of diabetes, accounting for approximately 90% of the nearly 537 million people living with diabetes worldwide [2].

In addition to these complications, T2DM leads to prolonged hyperglycemia, which adversely affects the nervous system, musculoskeletal structures, and cognitive functions [3]. As a result of this multidimensional involvement, individuals experience postural balance impairments in addition to functional limitations [4]. Balance problems are common in people with type 2 diabetes, and this condition is associated with an increased risk of falls [5,6,7]. Although balance disorders are most commonly associated with diabetic peripheral neuropathy, changes in postural control have also been reported in individuals with type 2 diabetes who do not have neuropathy [7]. In addition, T2DM has a greater impact on cognitive functions, particularly in areas such as attention, executive function, processing speed and memory [8,9]. The co-occurrence of these cognitive and motor impairments can contribute to a decline in functional status and independence in activities of daily living. Executive functions play a key role in planning and coordinating the complex behaviors required during daily activities. As most activities of daily living require the simultaneous execution of cognitive and motor tasks, impairments in these processes can negatively affect walking, balance, and functional status [10].

In the management of T2DM, lifestyle changes, especially physical activity and regular exercise, play an important role alongside pharmacological treatments [11]. Exercise has been shown to improve glycemic control, reduce HbA1c levels, and enhance muscle strength and functional status [3,12,13]. In this context, dual-task exercises stand out as an approach that simultaneously targets both cognitive and motor processes [14]. Dual tasking refers to an individual’s capacity to perform two different tasks simultaneously and typically involves the execution of a cognitive or motor secondary task accompanying a primary motor task. The main aim in this approach is to share attention between the two tasks [15,16].

Dual-task exercises have been studied in both healthy individuals and various patient groups. They have been reported to improve postural balance and reduce the risk of falls in healthy elderly individuals [17,18]. It has also been reported to have positive effects on gait, postural balance, and cognitive performance in neurological conditions such as mild cognitive impairment, dementia, Parkinson’s disease, multiple sclerosis, and stroke [19,20,21,22,23,24]. However, standardized protocols encompassing frequency, intensity, duration, type, volume, and progression principles for dual-task training have not yet been established [25].

Individuals with T2DM may present with multiple coexisting problems, including slowed cognitive processing and impaired executive functions, as well as loss of proprioception, reduced postural control, and an increased risk of falls [6]. Since daily activities frequently require the simultaneous performance of motor and cognitive tasks, the coexistence of these impairments may negatively affect dual-task performance in individuals with T2DM [10]. Therefore, approaches aimed at evaluating and improving dual-task performance may be particularly important in chronic conditions such as T2DM, as they may contribute to a better understanding of functional limitations and the development of more effective rehabilitation strategies. A review of the current literature highlights the need for studies evaluating the effects of dual-task training in individuals with T2DM and for detailed descriptions of applied exercise protocols in detail [26,27]. Accordingly, the present study was designed to investigate the effects of a dual-task exercise approach compared with standard single-task exercises administered under the supervision of a physiotherapist in individuals with T2DM.

## 2. Materials and Methods

### 2.1. Study Design

This study was a prospective, randomized controlled trial conducted at Artvin State Hospital between February 2025 and August 2025. The study included individuals diagnosed with type 2 diabetes mellitus (T2DM) by a specialist physician in accordance with the American Diabetes Association (ADA) guidelines. Participants were randomly assigned to the single-task exercise group, the dual-task exercise group, or the control group using a computer-generated randomization sequence. Allocation concealment was ensured by having the randomization sequence generated by an independent researcher who was not involved in participant recruitment or the assessment process. The randomization sequence was generated by an independent researcher who was not involved in participant selection or assessment. Participant enrolment was carried out by the research team, whilst group allocations were performed according to the prepared randomization sequence. Blinding was not implemented in this study because the nature of the intervention did not allow blinding of participants or therapists. The study was conducted in accordance with the Declaration of Helsinki and reported in accordance with the Consolidated Standards of Reporting Trials guidelines (see Supplementary Materials).

### 2.2. Participants

Individuals aged 40–65 years with a diagnosis of T2DM who were followed at Artvin State Hospital were included in the study.

The inclusion criteria were as follows: having a confirmed diagnosis of T2DM, ability to ambulate independently, willingness to participate in exercise at least two days per week as required by the study protocol (for the intervention groups), absence of cooperation or communication problems, and not having participated in a regular exercise program within the previous six months.

The exclusion criteria included the presence of T2DM-related macrovascular (e.g., cardiovascular disease, peripheral arterial disease, cerebrovascular disease) or microvascular (e.g., diabetic peripheral neuropathy, retinopathy, nephropathy) complications; orthopedic, neurological, or surgical conditions that would limit walking or exercise participation; diagnosed sensory loss; diagnosed mental disorders; cardiac, pulmonary, or systemic diseases at a level that would preclude exercise; visual impairments not correctable with glasses or contact lenses; and hearing loss not correctable with hearing aids. Participants whose training had to be discontinued due to the development of uncontrolled diabetes or hypertension during the exercise sessions were withdrawn from the intervention. Individuals with serious diabetes-related complications, including diabetic peripheral neuropathy, were excluded from the study in order to minimize potential factors that could independently affect balance performance.

### 2.3. Sample Size

To determine the sample size of the study, the G*Power—A General Power Analysis Program (Version 3.1) was used (Heinrich Heine University Düsseldorf, Germany). Based on data from a previous study conducted in a different population using the Timed Up and Go Test (TUG) as one of the outcome measures, the effect size was calculated as 0.71 (effect size = 0.7144695). With a Type I error rate of 0.05 (α = 0.05) and 95% statistical power, it was calculated that a minimum of 36 participants was required across three groups, with 12 participants per group [28]. However, given a potential dropout rate of 20%, 45 participants (15 per group) were initially recruited. The study was ultimately completed with 40 participants. In the dual-task paradigm used in this study, dual-task performance was derived from the TUG performed under single-task and dual-task conditions. Therefore, TUG represents the motor component of the dual-task assessment and was used as the basis for estimating the required sample size for this trial.

### 2.4. Ethical Considerations

The study was approved by the Non-Interventional Clinical Research Ethics Committee of İzmir Democracy University with decision number 2024/9-6. All participants were informed about the study, and written informed consent was obtained from those who agreed to participate. In addition, the study was registered in ClinicalTrials.gov under the registration number NCT06819371.

### 2.5. Assessments

Sociodemographic data were collected from all participants at baseline. All other assessments were conducted at the beginning of the study and at the end of the eight-week intervention period. The primary outcome of this study was dual-task performance. Secondary outcomes included balance, functional status, and cognitive function. Attendance at make-up sessions and adherence to the exercise program were recorded.

#### 2.5.1. Sociodemographic and Clinical Characteristics

Data regarding participants’ age, height, weight, body mass index (BMI), duration of diabetes, sex, educational level, employment status, dominant extremity, and smoking and alcohol consumption habits were recorded. In addition, the Charlson Comorbidity Index (CCI), which assesses comorbid conditions, was administered. This index was developed to estimate mortality risk, with scores ranging from 1 to 3, where 1 indicates low, and 3 indicates high mortality risk [29].

#### 2.5.2. Dual-Task Assessment

There is no single standardized test for evaluating dual-task performance. However, in the literature, dual-task performance is commonly assessed by adding an additional cognitive or motor task to gait, postural control, or balance tests. In the assessment process, performance on the primary task is first measured under single-task conditions, followed by concurrent performance with a secondary motor or cognitive task. Comparisons are then made between the scores obtained under single-task and dual-task conditions [30]. Commonly used secondary motor tasks in the literature include carrying a tray with a glass of water, picking up objects from different heights, pressing a button, or transferring an object from one hand to the other [31,32]. Common secondary cognitive tasks include forward and backward digit sequences, counting the days of the week forward or backward, category-based word generation, letter–color discrimination, arithmetic calculations, and verbal repetition [31,32,33]. In this study, TUG was used to assess dual-task performance. Participants first completed the TUG under single-task conditions, and the completion time was recorded (TUG–single task condition). Subsequently, the test was performed concurrently with a cognitive task requiring discrimination of the sound “a” from a series of spoken letters while walking (TUG–dual task condition). The duration of both conditions was recorded in seconds. The difference between performing the primary task alone and under dual-task conditions represents the dual-task effect. To quantify the performance change under dual-task conditions, the dual-task effect was calculated using the TUG completion times obtained in both conditions according to the following formula:

Dual-task effect = [(dual-task performance) − (single-task performance)]/(single-task performance)

#### 2.5.3. Assessment of Balance

Four Square Step Test (FSST): This test focuses on dynamic balance and assesses postural control during directional changes. For test administration, four equal squares are marked on the floor using sticks, rods, or tape. The squares are numbered 1–4 and explained to the participants. The participant is instructed to step between the squares in a predetermined sequence, then return to the starting position in the reverse order, without touching the floor markings. The time required to complete the test is recorded in seconds, and the best performance out of three trials is documented [34].

Balance Error Scoring System (BESS): This test evaluates postural control across different surface and stance conditions. Participants are asked to maintain double-leg, single-leg, and tandem stances for 20 s with their eyes closed on both stable and unstable surfaces. Prior to testing, conditions considered as errors are explained, and one practice trial is provided for each position. The assessment is conducted via video recording, and the score is determined by the number of errors. A higher error score indicates poorer postural control [35].

#### 2.5.4. Assessment of Functional Status

Timed Up and Go Test (TUG): A simple, commonly used test of walking speed and functional mobility. The participant stands up from a chair, walks to a point 3 m away, turns around, walks back, and sits down again. The time to complete the task is recorded in seconds, and the mean value of three trials is used as the final score [36]. The TUG was used for two distinct purposes in this study. First, TUG performed under single-task conditions (TUG-single task condition) was used as the baseline motor component of the dual-task paradigm and served as the reference for calculating dual-task performance outcomes, including TUG-dual task condition and the dual-task impact score. Second, the TUG was also analyzed separately as an indicator of functional mobility within the functional status domain. Although the same test was used, these analyses represent conceptually different constructs: dual-task performance versus functional mobility.

Functional Reach Test (FRT): This test is a practical method for assessing dynamic balance. The participant stands sideways next to a wall, extends the arm forward at 90° of shoulder flexion, and the starting position is marked. Without flexing the knees or taking a step, the participant reaches forward as far as possible, and the furthest point reached is marked. The distance between the two marked points is measured and recorded in centimeters [37].

#### 2.5.5. Assessment of Cognitive Functions

Montreal Cognitive Assessment (MoCA): The MoCA is a brief screening tool that takes approximately 10 min to administer and rapidly evaluates multiple cognitive domains. It assesses orientation, attention, memory, language, visuospatial abilities, and executive functions. The total score ranges from 0 to 30, with scores above 21 indicating normal cognitive function [38].

Digit Span Test: This test consists of two components—forward and backward digit span—and is used to assess attention and short-term memory. Participants are asked to repeat sequences of numbers presented at one-second intervals, in the same order for the forward span and in reverse order for the backward span. The number of digits in the longest correctly repeated sequence is recorded as the score [39].

Stroop Test: The Stroop test is used to evaluate executive functions, including simple and complex attention, information processing speed, inhibition, and resistance to interference. Various versions of the test exist; in this study, a three-stage version was employed. In the first stage (Stroop A), participants are asked to name the colors of the boxes; in the second stage (Stroop B), they are instructed to read the words as quickly as possible; and in the third stage (Stroop C), they are asked to name the color of the ink in which the words are printed. The completion time for each stage is recorded in seconds. For scoring purposes, the difference between the Stroop C and Stroop B completion times is calculated as the Stroop D score [40].

Controlled Word Association Test: This test assesses semantic and phonemic fluency and evaluates executive functions, including verbal fluency, mental flexibility, information processing speed, inhibition, and semantic organization. The test consists of two stages. In the first stage, participants are asked to generate as many words as possible within a specific semantic category (animal names) in one minute. In the second stage, participants are asked to produce words beginning with K, A, and S, each for 1 min. The number of animal names generated is recorded as the semantic fluency score, while the total number of words produced for the letters K, A, and S constitutes the phonemic fluency score [41].

### 2.6. Interventions

After baseline assessments were completed, participants randomly allocated to three groups received a planned exercise program in addition to their routine medical treatment. The intervention was conducted twice weekly for 8 weeks. All sessions were carried out individually in an isolated room under the supervision of a physiotherapist. The exercise program was designed in accordance with the exercise prescription principles recommended by the ACSM for adults with T2DM [42].

Each exercise session began with a standard warm-up protocol consisting of active range-of-motion exercises targeting major joints. The warm-up included head rotation; cervical flexion–extension; trunk flexion, extension, and rotation; ankle dorsiflexion–plantarflexion; knee flexion–extension; and hip flexion, extension, and abduction. Each movement was performed for one set of ten repetitions. At the end of each session, a general cool-down protocol consisting of stretching exercises targeting large muscle groups, including the shoulders, hips, knees, ankles, and the lateral flexors of the trunk was applied.

Between the warm-up and cool-down protocols, both intervention groups performed a common, progressively structured balance exercise program over an eight-week period. The progression of the balance exercises was planned at 2-week intervals.

During the first two weeks, exercises included standing with feet apart and together on a firm surface, combined with saccadic eye movements, tandem stance, single-leg stance, and forward, backward, lateral, and tandem walking. In weeks 3–4, eye-tracking exercises were added to the program; balance exercises were progressed by altering foot positions on firm and compliant surfaces, and walking on the toes and heels was introduced. In weeks 5–6, gaze stabilization exercises were initiated; standing and single-leg stance exercises were further progressed using different arm positions (posterior, anterior, and diagonal), and backward tandem walking was added. In the final phase of the program (weeks 7–8), eye–head coordination exercises were performed, along with direction changes on a compliant surface, figure-of-eight walking, and tandem walking exercises.

In the balance exercise program, eye exercises were performed for 1 set of 10 repetitions, standing exercises were held for 30 s per condition, and walking exercises were performed over a 3 m distance for 2 laps.

Single-Task Exercise Group: Participants in this group focused solely on the balance exercises common to both intervention groups throughout the exercise sessions. No additional motor or cognitive tasks were provided during the balance exercises.

Dual-Task Exercise Group: Participants randomized to this group performed balance exercises concurrently with a secondary task throughout the exercise sessions. The content, duration, number of repetitions, and sets of the warm-up, cool-down, and balance exercises were identical to those of the single-task exercise group. However, in this group, cognitive tasks were provided as the secondary task during balance exercises. Each week, tasks targeting at least two different cognitive domains were selected, and progression from simple to more complex tasks was adopted based on individual performance to minimize learning effects. The selection of cognitive tasks was guided by examples reported in the literature [43,44,45]. In the dual-task exercise group, balance exercises were performed simultaneously with tasks targeting cognitive domains including attention, memory, executive functions, visuoperceptual functions, and processing speed. Cognitive supplementary tasks included sequential subtraction, verbal fluency, word/sentence recall, response tasks, counting tasks, and similarity/difference discrimination tasks. The cognitive tasks were structured from simple to complex according to participants’ performance levels. Task complexity, number of stimuli, and cognitive load were systematically modified to reduce learning effects. Detailed examples of the secondary cognitive tasks used in the dual-task exercise group are provided in Supplementary Table S1.

Participants randomized to the control group received no exercise intervention beyond their routine medical treatment and standard recommendations. A summary of the intervention protocol is presented in Table 1 to improve clarity and reproducibility.

Attendance and adherence to the intervention were monitored throughout the study. If participants were unable to attend a scheduled session, a make-up session was arranged within the same week to maintain program continuity. As a result, all participants who completed the study attended the full 16 exercise sessions, and both exercise groups received the intended intervention dose. Participants who missed two consecutive sessions and were unable to attend make-up sessions (for example, due to unsuitable work circumstances or moving to another city) were withdrawn from the study. No adverse events or safety concerns were reported during the intervention period.

### 2.7. Statistical Analysis

Statistical analyses were performed using IBM SPSS Statistics for Windows, Version 26.0 (IBM Corp., Armonk, NY, USA). The normality of continuous variables was assessed using the Shapiro–Wilk test, which indicated that the data were not normally distributed. Therefore, nonparametric statistical methods were used for the analysis. Descriptive statistics were presented as median and first–third quartile values (Q1/Q3). The chi-square test was used to compare categorical variables. Baseline measurements and between-group change scores (Δ) were compared using the Kruskal–Wallis test. Within-group comparisons were conducted using the Wilcoxon signed-rank test. Effect sizes (r) were calculated using Z values obtained from the Wilcoxon signed-rank test. To identify the source of significant differences between groups, post hoc pairwise comparisons were performed using the Mann–Whitney U test with Bonferroni correction. This nonparametric method was selected given the non-normal distribution of the data and the relatively small sample size. Statistical significance was set at *p* < 0.05. During the preparation of this text, the authors used an artificial intelligence-based language model (ChatGPT, OpenAI, San Francisco, CA, USA) to improve language editing and increase the clarity and readability of the text.

## 3. Results

A total of 68 individuals were assessed for eligibility. Participants were randomly assigned to study groups using a computer-generated random order. Forty-five individuals who met the inclusion criteria were divided into three groups: a dual-task exercise group (DTEG; *n* = 15), a single-task exercise group (STEG; *n* = 15), and a control group (CG; *n* = 15). Initial assessments were completed for all participants in each group.

**Figure 1:**
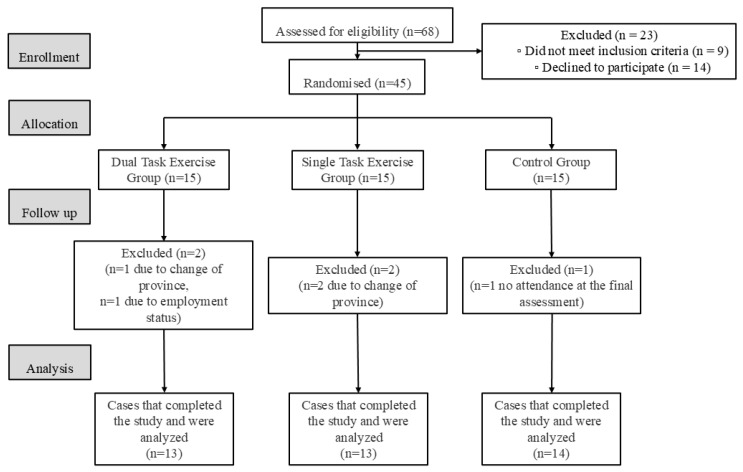
Study flow diagram.

During the study period, one participant from the DTEG and two participants from the STEG withdrew voluntarily due to relocation. In addition, one DTEG participant was unable to complete the study due to work-related constraints. In the CG, one participant was excluded for failing to attend the final assessment. Final evaluations were conducted at the end of the eight-week period for the remaining participants. Consequently, the study was completed with 13 participants in the DTEG, 13 in the STEG, and 14 in the CG, yielding a total of 40 individuals with T2DM included in the analysis. The study flow diagram is presented in Figure 1.

The demographic and clinical characteristics of the groups are presented in Table 2. No statistically significant differences were found between the groups in age, height, weight, BMI, gender, educational status, employment status, dominant side, smoking and alcohol use, or presence of comorbidities (*p* > 0.05).

Table 3 presents the pre- and post-intervention outcomes for balance, functional status, cognitive function, and dual-task performance for STEG, DTEG, and CG. Baseline values were generally comparable among the groups. After the eight-week intervention, both exercise groups demonstrated greater improvements across multiple outcome measures than the control group (Table 3).

For the primary outcome, dual-task performance, both groups showed significant improvements in the TUG single-task condition following the intervention. The DTEG demonstrated a very large and clinically meaningful effect size (r = 0.90), while the STEG exhibited a large effect size (r = 0.78). Similarly, very large effect sizes were observed in both groups for the TUG dual-task condition. In contrast, the impact score showed moderate effect sizes in both the STEG (r = 0.36) and the DTEG (r = 0.42).

In the present study, balance and functional status parameters demonstrated statistically significant improvements in both STEG and DTEG. In addition, cognitive functions also showed significant improvements. Effect size analysis further indicated that the magnitude of these improvements ranged from moderate to very large (r = 0.36–0.91), suggesting clinically meaningful effects. In contrast, in the control group, statistically significant changes were observed only in the backward range and Stroop D measures.

To evaluate the relative superiority between groups, pre- to post-intervention change (Δ) scores were calculated and compared for all parameters. For the primary outcome measure, dual-task performance, statistically significant between-group differences were observed in the change scores for the TUG single-task condition (*p* < 0.001) and TUG dual-task condition (*p* < 0.001). Post hoc analyses showed that both exercise groups exhibited greater improvements than the control group.

For the primary outcome, dual-task performance, as well as balance, functional status, and cognitive outcomes, significant differences were observed among the groups (Table 4). In balance intergroup comparisons were significant for the total BESS score (*p* < 0.001) and the FSST (*p* < 0.001). Post hoc analyses indicated that these differences were primarily attributable to changes between the exercise groups and the control group.

In terms of functional status, statistically significant between-group differences were observed in the change scores for the TUG (*p* < 0.001) and the FRT (*p* < 0.001). Both DTEG and STEG demonstrated greater improvement than the CG.

Regarding cognitive functions, significant between-group differences were found in change scores for the DTEG MoCA total score (*p* < 0.001) greater than STEG and CG. Also, delayed recall (*p* < 0.001), forward range (*p* < 0.001), backward range (*p* < 0.001), Stroop D (*p* < 0.001), semantic fluency (*p* = 0.005), and phonemic fluency (*p* < 0.001). Post hoc analyses revealed that these differences were largely driven by greater improvements in the DTEG and STEG compared with the CG. In contrast, no significant between-group differences were observed in the visuospatial–executive, naming, attention, concentration, language, abstract thinking, or orientation subtests (*p* > 0.05).

## 4. Discussion

The findings of the present study demonstrate that both single-task and dual-task exercise interventions have positive effects on cognitive function, balance, and functional status. The main finding of the study was that both interventions improved various outcomes compared to the control group. However, our findings do not demonstrate that dual-task training was statistically superior to single-task training in most measurements. Therefore, the results should be interpreted cautiously and do not indicate equivalence between the two approaches. A review of the literature reveals inconsistent findings regarding the effects of single-task and dual-task exercise approaches on cognitive functions, balance, and functional status in individuals with T2DM. While some studies report that dual-task exercises may provide additional benefits for cognitive and motor outcomes, others suggest that there is no significant superiority of the dual-task approach over the single-task approach [46,47,48,49,50].

In a study by Kraiwong et al., an eight-week physical–cognitive combined training program improved balance, lower extremity muscle strength, and cognitive outcomes [46]. In contrast, Balcı et al. found no significant differences in gait speed and balance outcomes among single-task, dual-task, and sequential physical–cognitive protocols in healthy older adults [47]. Furthermore, a meta-analysis demonstrated that exercise-based interventions significantly improved global cognitive performance in older adults with T2DM; however, no definitive conclusion could be drawn regarding the most effective type of exercise [48]. Similarly, a systematic review investigating dynamic balance in individuals with T2DM reported that exercise generally improves balance, yet no consensus exists regarding the optimal exercise prescription [49]. Studies conducted in different populations have also shown that both single-task and dual-task exercise approaches can yield meaningful benefits, but dual-task training is not consistently superior to single-task interventions. For example, a meta-analysis of individuals with Parkinson’s disease reported no clear superiority of dual-task training over single-task training for balance and gait performance [50]. The findings of the present study are consistent with previous research suggesting that different exercise modalities may have comparable effects on cognitive and motor performance in individuals with T2DM.

The primary outcome measure of our study is dual-task performance. Secondary outcomes such as balance, functional status, and cognitive function should therefore be interpreted as exploratory findings. When examining dual-task performance, the primary outcome measure of this study, an improvement trend was observed in TUG performance accompanied by cognitive tasks in both exercise groups. However, no statistically significant difference was found in the dual-task impact score (DT impact score) normalized to the single-task condition in within-group analyses. In between-group comparisons, it was determined that the exercise groups showed greater improvements in TUG performance compared to the control group in both single-task and dual-task conditions. However, these improvements were not reflected in the dual-task impact score, and no significant difference was found between the groups in terms of this parameter.

These study results demonstrate that both exercise approaches can improve task performance under cognitive load. However, the lack of a significant change in the dual-task impact score, normalized to single-task performance, suggests that the improvements may have largely stemmed from changes in overall motor performance. This indicates that more specific or longer-term interventions may be needed to reduce dual-task interference that occurs during the simultaneous execution of motor and cognitive tasks.

Another key finding of this study is that physical exercise plays a fundamental role in improving cognitive and postural outcomes in individuals with T2DM. However, the addition of cognitive load to physical exercise (dual-task training) did not provide statistically significant additional short-term benefits compared with standard single-task exercise interventions for most outcomes. However, these findings should not be interpreted as proof of equivalence between the two exercise approaches. Instead, the results demonstrate that both single-task and dual-task exercise interventions can contribute to improvements in motor, cognitive, and functional outcomes.

It has been shown that individuals with T2DM exhibit significantly poorer performance in core cognitive domains, including attention, executive functions, memory, and processing speed, compared with healthy individuals [9]. In individuals with T2DM who present with cognitive impairment, non-pharmacological interventions play a crucial role in supporting cognitive function. In particular, cognitive training and exercise have been emphasized as important strategies for cognitive support [51]. In a randomized controlled trial involving individuals aged 60 years or older with T2DM and mild cognitive impairment (MCI), the effects of Tai Chi on cognitive function were investigated. The Tai Chi group demonstrated greater improvements in cognitive performance compared with walking and control groups. The authors attributed this superiority to the multicomponent nature of Tai Chi, which incorporates not only aerobic elements but also balance and strength components, as well as its potential neurotrophic effects [52]. Accordingly, it can be suggested that the balance exercises included in both intervention groups in the present study may have activated cognitive-supportive mechanisms similar to those proposed for Tai Chi, thereby contributing to the observed improvements in cognitive function.

In the present study, single-task exercises were found to produce significant improvements, particularly in executive functions and memory performance, while also supporting attention and working memory processes. These findings are consistent with previous studies reporting that physical exercise enhances cerebral blood flow and oxygenation, thereby improving neurovascular function [53]. Moreover, regular physical activity has been shown to increase levels of brain-derived neurotrophic factor (BDNF), which supports synaptic plasticity and plays a critical role in learning and memory processes [54]. These mechanisms may underlie the cognitive improvements observed in this study.

Dual-task exercises, another intervention modality examined in this study, were found to provide broader cognitive benefits by simultaneously activating cognitive and motor systems. Following the eight-week intervention, significant improvements were observed in attention, working memory, executive functions, memory, and language domains. These findings suggest that the concurrent execution of motor and cognitive tasks may enhance functional connectivity in the prefrontal cortex, thereby increasing the efficiency of cognitive processes. Indeed, previous research has reported that motor–cognitive exercises increase prefrontal activity and improve inhibition, cognitive flexibility, and information processing speed [55]. Furthermore, when cognitive function was examined, the Control group showed statistically significant changes on the Stroop D and backward number sequence tests. However, the change in backward digit span reflected a decline rather than an improvement. This finding may be related to normal fluctuations in cognitive performance or variability associated with repeated testing in a small sample. In addition, the change observed in the Stroop test may be related to a learning effect associated with repeated testing rather than to improvement resulting from a genuine intervention. It is well known that cognitive assessments such as the Stroop test are susceptible to learning effects when administered repeatedly [56].

Functional status is defined as an individual’s ability to perform daily activities such as walking and stair climbing without excessive fatigue or difficulty [57]. Regarding functional outcomes, both single-task and dual-task exercise interventions resulted in significant improvements in the Timed Up and Go Test and the Functional Reach Test. These findings indicate that exercise effectively enhances functional status. The observed improvements in functional status in both exercise groups may be attributable to distinct neurophysiological mechanisms that produce similar outcomes. In the single-task exercise group, focusing exclusively on balance and postural control may have led to increased lower-extremity muscle strength, enhanced proprioceptive feedback, and improved functional gait. Previous studies have similarly demonstrated that such exercises directly influence functional status by improving muscle strength and postural stability [58]. In the dual-task exercise group, the addition of cognitive tasks may have enhanced neuromuscular control by increasing attentional engagement, activating executive functions, and facilitating concurrent motor planning. This may explain the more pronounced improvements in motor skills associated with executive control, such as direction changes, balance maintenance, and pace regulation. The combined execution of motor and cognitive tasks has been shown to strengthen prefrontal cortex activity, thereby enhancing cognitive flexibility and executive functions [55].

The functional gains observed in both exercise groups may be attributed to exercise-induced neuromuscular adaptation processes. It has been shown that eight weeks of regular exercise support neuromuscular adaptation in muscle strength, balance control, and motor coordination, which, in turn, enhance postural stability and functional status [53]. Accordingly, physical activity may indirectly influence cognitive processes by increasing attention and reaction time, thereby facilitating faster, more controlled motor responses.

Balance problems are closely associated with falls in daily life, and this issue is more pronounced in individuals with diabetes. Compared with healthy individuals, people with diabetes have been reported to have a higher risk of fall-related injuries during walking [59]. Another problem related to balance impairments in individuals with diabetes is reduced postural stability [60]. In the present study, initial findings indicated significant improvements in static balance performance in both exercise groups. However, adding cognitive tasks to balance exercises did not result in a marked difference in static balance outcomes. When dynamic balance outcomes were examined, improvements in direction changes and postural control during movement were observed in both groups. These findings suggest that the applied exercise programs may have strengthened dynamic balance strategies. Balance is a multidimensional construct that requires not only motor control but also effective use of attention, planning, and cognitive resources. Visual, vestibular, and somatosensory inputs are integrated by the central nervous system to maintain balance [61]. The findings of this study suggest that both exercise approaches stimulate sensorimotor integration processes supporting postural control in different ways and contribute to improvements in balance. The reduction in Balance Error Scoring System error scores may be associated with enhanced proprioceptive sensitivity, strengthened muscle spindle feedback, and improved coactivation of postural muscles. These cognitive optimizations observed in static balance may also have positively influenced dynamic balance tasks.

The maintenance of postural control under both static and dynamic conditions depends on the combined functioning of automatic and cognitive mechanisms. Moreover, postural control is sustained not only by reflexive processes but also through coordinated activity of cortical and subcortical networks; impairments in divided attention have been shown to disrupt coordination [20]. Accordingly, dual-task exercises may reduce the cognitive resources required to maintain postural control, making balance more automatic and less cognitively demanding. This mechanism may partly explain why dual-task exercises yielded outcomes similar to those of single-task exercises in the present study.

One of the strengths of this study is its emphasis on the fundamental role of structured physical activity in planning exercise-based rehabilitation for individuals with T2DM, while also considering dual-task exercises as a complementary approach that can be tailored to individual functional needs. This perspective contributes to the development of feasible and sustainable exercise programs in clinical practice.

Several limitations should be considered when interpreting the findings of this study. First, individuals with diabetic complications such as diabetic peripheral neuropathy were excluded from the study. While this exclusion allows for a clearer assessment of the intervention’s effects, it may limit the generalizability of the findings to the broader population of individuals with type 2 diabetes. Future studies including individuals with diabetic complications are needed to determine whether similar effects are observed in more heterogeneous clinical populations. Second, participants’ footwear was not standardized during balance assessments. Footwear characteristics have been shown to affect posture and dynamic balance during functional activities [62,63]. Therefore, the lack of control over footwear may have led to variability in balance performance. Future studies should standardize footwear conditions or record them to minimize this potential source of variability. Finally, the 8-week follow-up period is relatively short; longer follow-ups may yield more information on the sustainability of treatment effects.

Future studies may further strengthen the evidence by incorporating more objective assessment and intervention approaches. For example, the use of technological assessment tools, such as balance platforms and three-dimensional motion analysis systems, can enable a more objective and precise evaluation of postural stability and balance strategies. Similarly, in addition to clinical cognitive tests, neuroimaging methods such as functional magnetic resonance imaging (fMRI) could be incorporated to better elucidate underlying mechanisms. Furthermore, implementing cognitive tasks in dual-task exercises via computer-assisted or digital platforms may facilitate standardization of cognitive stimulation and individualized adjustment of task difficulty. This approach could enable a more objective assessment of the cognitive components of exercise and allow digital monitoring of progression.

## 5. Conclusions

In conclusion, this study demonstrated that regular exercise in individuals with T2DM resulted in significant improvements in cognitive function, balance, and functional status. Both single-task and dual-task exercise protocols produced similar levels of positive effects compared to the control group. These findings underscore that regular exercise is a fundamental component in improving cognitive and motor performance in individuals with T2DM.

## Figures and Tables

**Table 1:** Summary of the exercise intervention protocol.

Group	Session Frequency	Session Duration	Program Duration	Content	Progression	Supervision
STEG	2 sessions/week	40 min/session	8 weeks	Warm-up; Balance and gait training; Cool-down	Progressive increase in task difficulty every two weeks	Supervised by a physiotherapist
DTEG	2 sessions/week	40 min/session	8 weeks	Warm-up; Balance and gait training performed under dual-task conditions (additional tasks covering at least two different cognitive areas each week); Cool-down	Balance and gait task difficulty progressively increased every two weeks, while cognitive task complexity was adjusted according to participant performance	Supervised by a physiotherapist
CG	-	-	8 weeks	No structured exercise	-	-

**Table 2:** Demographic Characteristics of Individuals Included in the Study.

Variables	STEG (*n* = 13)	DTEG (*n* = 13)	CG (*n* = 14)	*p* Value
Age (years)	58 (53.0/61.5)	55 (50.0/60.5)	56.5 (53.0/61.3)	0.902
Height (cm)	171 (160.0/177.5)	166 (159.5/175.0)	169 (161.8/176.3)	0.872
Weight (kg)	84 (76.0/90.0)	80 (70.5/84.0)	86.5 (76.8/96.0)	0.671
BMI (kg/m^2^)	28.4 (26.4/32.5)	28.4 (25.9/29.5)	29.7 (27.0/33.5)	0.480
Diabetes Year	7 (5.0/11.5)	8 (5.5/14.5)	6.5 (3.8/10.3)	0.711
	*n* (%)	*n* (%)	*n* (%)	
Gender				
Female	6 (46.2%)	8 (61.5%)	6 (42.9%)	0.590
Male	7 (53.8%)	5 (38.5%)	8 (57.1%)	
Educational Status				
Primary education	2 (15.4%)	2 (15.4%)	2 (14.3%)	0.646
High school	4 (30.8%)	4 (30.8%)	4 (28.6%)	
Associate degree	2 (15.4%)	2 (15.4%)	1 (7.1%)	
Bachelor’s degree	4 (30.8%)	4 (30.8%)	5 (35.7%)	
Postgraduate	1 (7.7%)	1 (7.7%)	2 (14.3%)	
Work Status				
Working	8 (61.5%)	8 (61.5%)	11 (78.6%)	0.778
Not working	4 (30.8%)	3 (23.1%)	2 (14.3%)	
Retired	1 (7.7%)	2 (15.4%)	1 (7.1%)	
Dominant side				
Right	13 (100%)	12 (92.3%)	13 (92.9%)	0.601
Left	0 (0%)	1 (7.7%)	1 (7.1%)	
Cigarette Use				
Exists	2 (15.4%)	1 (7.7%)	3 (21.4%)	0.134
No	5 (38.5%)	10 (76.9%)	4 (28.6%)	
Has quit smoking	6 (46.2%)	2 (15.4%)	7 (50%)	
Alcohol use				
Exists	3 (23.1%)	1 (7.7%)	3 (21.4%)	0.523
No	10 (76.9%)	12 (92.3%)	11 (78.6%)	
Comorbidity				
1	7 (53.8%)	9 (69.2%)	7 (50%)	0.570
2	6 (46.2%)	4 (30.8%)	7 (50%)	

Data are presented as median (IQR) or *n* (%); STEG: single-task exercise group; DTEG: dual-task exercise group; CG: control group; BMI: body mass index; Kruskal–Wallis test and Chi-square test were used for group comparisons; *p* < 0.05.

**Table 3:** Comparison of pre- and post-treatment results between single-task, dual-task, and control groups.

Variable	STEG Pre	STEG Post	*p*	DTEG Pre	DTEG Post	*p*	CG Pre	CG Post	*p*
Dual task performance									
TUG (single-task condition)	10.0 (9.5/11.5)	10.0 (8.5/10.5)	**0.005**	11.0 (11.0/13.0)	9.0 (8.5/11.0)	**<0.001**	11.0 (11.0/12.0)	10.25 (9.75/12.0)	0.137
TUG (dual-task condition)	14.0 (14.0/15.0)	12.0 (11.0/13.0)	**<0.001**	15.0 (15.0/16.5)	12.0 (10.0/13.0)	**<0.001**	13.0 (13.0/14.0)	13.0 (12.0/14.25)	0.942
DT impact score	25.0 (13.3/40.0)	20.0 (12.5/36.65)	0.196	22.2 (12.65/37.35)	18.1 (11.8/33.3)	0.133	19.05 (15.03/27.2)	26.54 (16.0/34.05)	0.084
Balance									
BESS									
BESS total	25.0 (19.0/30.5)	15.0 (11.5/20.0)	**0.001**	24.0 (21.0/26.0)	17.0 (13.0/18.0)	**<0.001**	26.0 (21.0/27.0)	24.5 (20.0/27.0)	0.126
FSST	11.0 (10.0/13.25)	9.5 (8.75/11.75)	**<0.001**	11.0 (9.75/12.0)	9.0 (8.5.0/10.0)	**0.002**	10.5 (10.0/11.25)	10.25 (10.0/12.0)	0.928
Functional status									
TUG (functional mobility)	10.0 (9.5/11.5)	10.0 (8.5/10.5)	**0.005**	11.0 (11.0/13.0)	9.0 (8.5/11.0)	**<0.001**	11.0 (11.0/12.0)	10.25 (9.75/12.0)	0.137
FRT	30.0 (27.0/36.5)	32.0 (29.5/38.0)	**0.002**	28.0 (24.5/40.0)	31.0 (26.5/42.5)	**<0.001**	32.0 (29.25/35.5)	32.0 (28.0/34.25)	0.321
Cognitive functions									
MoCA total	23.0 (21.5/25.5)	26.0 (24.0/28.5)	**<0.001**	23.0 (22.0/26.5)	29.0 (26.5/30.0)	**<0.001**	23.5 (21.75/25.25)	23.5 (22.0/26.25)	0.248
Visual/Executive	1.0 (1.0/2.5)	3.0 (2.5/4.0)	**0.046**	2.0 (2.0/2.0)	4.0 (3.0/5.0)	**0.014**	1.0 (1.0/2.25)	1.5 (0.0/3.0)	0.317
Naming	3.0 (2.0/3.0)	3.0 (3.0/3.0)	**0.046**	3.0 (2.0/3.0)	3.0 (2.5/3.0)	0.157	3.0 (3.0/3.0)	3.0 (3.0/3.0)	1.0
Attention	2.0 (1.50/2.0)	2.0 (2.0/2.0)	0.157	2.0 (1.0/2.0)	2.0 (2.0/2.0)	**0.023**	2.0 (1.75/2.0)	2.0 (1.75/2.0)	1.0
Concentration	3.0 (3.0/4.0)	4.0 (3.0/4.0)	0.317	4.0 (3.0/4.0)	4.0 (3.0/4.0)	0.157	4.0 (3.0/4.0)	4.0 (3.0/4.0)	0.317
Language	3.0 (2.0/3.0)	3.0 (2.5/3.0)	0.157	2.0 (2.0/3.0)	3.0 (2.5/3.0)	**0.046**	2.0 (2.0/3.0)	2.0 (2.0/3.0)	1.0
Abstract Thinking	1.0 (1.0/2.0)	2.0 (1.0/2.0)	**0.046**	2.0 (1.5/2.0)	2.0 (2.0/2.0)	0.102	2.0 (2.0/2.0)	2.0 (2.0/2.0)	0.317
Delayed Recall	1.0 (1.0/2.5)	3.0 (2.5/4.0)	**0.003**	2.0 (2.0/2.0)	4.0 (3.0/5.0)	**<0.001**	1.0 (1.0/2.25)	1.5 (0.0/3.0)	0.739
Orientation	6.0 (6.0/6.0)	6.0 (6.0/6.0)	**0.317**	6.0 (6.0/6.0)	6.0 (6.0/6.0)	0.317	6.0 (6.0/6.0)	6.0 (6.0/6.0)	1.0
Forward Range	25.0 (19.0/30.5)	15.0 (11.5/20.0)	**0.048**	24.0 (21.0/26.0)	14.0 (12.0/17.5)	**<0.001**	26.0 (21.0/27.75)	24.5 (20.0/27.0)	0.102
Backward Range	3.0 (3.0/4.0)	4.0 (3.0/4.0)	**0.025**	4.0 (3.0/4.0)	4.0 (3.5/5.0)	**0.007**	4.0 (4.0/5.0)	4.0 (3.0/4.0)	**0.014**
Stroop D	65.0 (51.0/79.0)	55.0 (37.5/64.0)	**<0.001**	45.0 (40.0/56.0)	35.0 (31.5/45.0)	**<0.001**	53.0 (44.75/70.25)	53.0 (44.75/67.5)	**0.013**
Semantic fluency	19.0 (16.0/23.0)	21.0 (18.5/25.0)	**<0.001**	23.0 (17.5/26.5)	24.0 (21.0/28.5)	**<0.001**	19.5 (17.0/23.25)	20.5 (16.75/24.0)	0.600
Phonemic fluency	35.0 (23.5/38.5)	37.0 (26.5/42.5)	**<0.001**	39.0 (36.5/52.0)	45.0 (42.5/57.0)	**<0.001**	43.5 (25.0/51.75)	43.5 (26.75/48.25)	0.617

BESS: Balance Error Scoring System, FSST: Four Square Step Test, TUG: Time Up and Go Test, FRT: Functional Reach Test, MoCA: Montreal Cognitive Assessment. Values are presented as median (interquartile range). TUG performed under single-task conditions represents the motor component of the dual-task paradigm and was also analyzed separately as an indicator of functional mobility. Bold values indicate statistically significant differences (*p* < 0.05).

**Table 4:** Between-group comparison of change scores (Δ) in balance, functional, cognitive, and dual-task outcomes.

Variables	STEG	DTEG	CG	*p* *
Dual task performance				
TUG (single-task condition)	−1.0 (−1.0/0.0) ^a^	−2.0 (−2.0/−1.0) ^a^	−0.50 (−1.0/0.13) ^b^	<0.001 *
TUG (dual-task condition)	−1.0 (−2.0/−1.0) ^a^	−2.0 (−3.50/−2.0) ^a^	0.0 (−1.0/1.0) ^b^	<0.001 *
DT impact score	−6.90 (−10.40/2.20)	−8.90 (−11.25/−0.10)	9.01 (−6.15/13.40)	0.111
Balance				
BESS total	−10.0 (−11.0/−6.0) ^a^	−9.0 (−10.0/−7.50) ^a^	−1.0 (−3.0/−0.75) ^b^	<0.001 *
FSST	−2.0 (−1.0/0.5) ^a^	−2.0 (−1.50/−1.0) ^a^	−0.25 (0.0/−0.62) ^b^	<0.001 *
Functional status				
TUG (functional mobility)	−1.0 (−1.0/0.0) ^a^	−2.0 (−2.0/−1.0) ^a^	−0.50 (−1.0/0.13) ^b^	<0.001 *
FRT	2.0 (1.0/2.0) ^a^	2.0 (2.0/3.0) ^a^	0.0 (−1.25/1.0) ^b^	<0.001 *
Cognitive functions				
MoCA total	2.0 (1.50/4.0) ^a^	4.0 (3.0/5.0) ^b^	0.0 (−0.25/1.0) ^a^	<0.001 *
Visual/Executive	0.0 (0.0/1.0)	0.0 (0.0/1.0)	0.0 (0.0/0.0)	0.077
Naming	0.0 (0.0/1.0)	0.0 (0.0/0.0)	0.0 (0.0/0.0)	0.087
Attention	0.0 (0.0/0.0)	0.0 (0.0/1.0)	0.0 (0.0/0.0)	0.090
Concentration	0.0 (0.0/0.0)	0.0 (0.0/0.0)	0.0 (0.0/0.0)	0.738
Language	0.0 (0.0/0.0)	0.0 (0.0/1.0)	0.0 (0.0/0.0)	0.087
Abstract thinking	0.0 (0.0/1.0)	0.0 (0.0/0.50)	0.0 (0.0/0.0)	0.308
Delayed recall	2.0 (1.0/2.0) ^a^	2.0 (1.0/3.0) ^a^	0.0 (−1.0/1.0) ^b^	<0.001 *
Orientation	0.0 (0.0/0.0)	0.0 (0.0/0.0)	0.0 (0.0/0.0)	0.575
Forward range	−10.0 (−11.0/−6.0) ^a^	−9.0 (−10.0/−7.50) ^a^	−1.0 (−1.25/0.0) ^b^	<0.001 *
Backward range	0.0 (0.0/0.0) ^a^	1.0 (0.0/1.0) ^a^	−1.0 (−2.0/1.0) ^b^	<0.001 *
Stroop D	−12.0 (−15.50/−7.0) ^a^	−10.0 (−21.50/−5.50) ^a^	1.0 (−1.25/1.25) ^b^	<0.001 *
Semantic fluency	2.0 (1.0/3.0) ^a^	2.0 (1.0/3.0) ^a^	0.0 (−1.0/1.25) ^b^	0.005 *
Phonemic fluency	3.0 (2.0/6.0) ^a^	5.0 (3.50/6.50) ^a^	1.0 (−1.25/1.25) ^b^	<0.001 *

BESS: Balance Error Scoring System, FSST: Four Square Step Test, TUG: Time Up and Go Test, FRT: Functional Reach Test, MoCA: Montreal Cognitive Assessment Scale, * Kruskal–Wallis test; post hoc pairwise comparisons were performed using Mann–Whitney U test with Bonferroni correction. Values are presented as median (interquartile range). Superscript letters (a, b) indicate significant differences between groups (*p* < 0.05). Values sharing the same letter are not significantly different. TUG performed under single-task conditions represents the motor component of the dual-task paradigm and was also analyzed separately as an indicator of functional mobility. Bold values indicate statistically significant differences (*p* < 0.05).

## Data Availability

The data presented in this study can be obtained from the corresponding author upon request. The data are not publicly available due to confidentiality and ethical restrictions.

## Supplementary Materials

The following supporting information can be downloaded at: https://www.mdpi.com/article/10.3390/jcm15072761/s1, CONSORT 2010 checklist of information to include when reporting a randomised trial; Supplementary Table S1. Examples of additional cognitive tasks given to the dual-task exercise group.

## Abbreviations

The following abbreviations are used in this manuscript:

BESS	Balance Error Scoring System
CG	Control Group
DTEG	Dual Task Exercise Group
FRT	Functional Reach Test
FSST	Four Square Step Test
MoCA	Montreal Cognitive Assessment Scale
STEG	Single Task Exercise Group
T2DM	Type 2 Diabetes Mellitus
TUG	Time Up and Go Test
